# Copeptin, pro-atrial natriuretic peptide and pro-adrenomedullin as markers of hypoxic stress in patients with obstructive sleep apnea—a prospective intervention study

**DOI:** 10.1186/s12931-021-01704-0

**Published:** 2021-04-20

**Authors:** Meropi Karakioulaki, Peter Grendelmeier, Werner Strobel, Thomas Schmid, Kathleen Jahn, Leticia Grize, Michael Tamm, Daiana Stolz

**Affiliations:** 1 Clinic of Respiratory Medicine and Pulmonary Cell Research, University Hospital of Basel, Petersgraben 4, 4031 Basel, Switzerland; 2 Privaltklinik Obach, Solothurn, Switzerland

**Keywords:** OSA, CPAP, ProADM, Copeptin, ProAMP

## Abstract

**Study Objectives:**

Obstructive sleep apnea (OSA) might lead to oxidative stress, inflammation and elevated circulating copeptin, proANP and proADM levels. We aimed to evaluate whether the levels of these prohormones are higher in patients with OSA and whether they might change under continuous positive airway pressure (CPAP) therapy, serving as potential proxies for the diagnosis and therapy-response in OSA.

**Methods:**

A total of 310 patients with suspicion of OSA were recruited. Screening for OSA was performed using overnight pulse oximetry followed by polygraphy and a venous puncture in the morning. All patients diagnosed with OSA underwent CPAP adaptation. A venous puncture was conducted in the night before CPAP and in the following morning. At 1 and 6 months of treatment, polygraphy was performed, followed by a venous puncture in the morning. In the acquired blood, copeptin, proANP and proADM levels were measured.

**Results:**

We analyzed 232 patients with OSA and 30 patients without OSA. Our results indicated that only copeptin levels differed significantly among patients with and without OSA at baseline. In OSA patients, the levels of proADM significantly changed after 1 and 6 months on CPAP therapy, when compared to baseline (p < 0.001 and p = 0.020). Additionally, proANP levels significantly decreased after 12 h on CPAP therapy, as compared to baseline levels (p < 0.001).

**Conclusions:**

Copeptin is significantly associated with the presence of OSA. ProANP levels might serve as a potential proxy for the acute response to non-invasive ventilation (12 h), while proADM reflects the long-term response (1 and 6 months).

**Supplementary Information:**

The online version contains supplementary material available at 10.1186/s12931-021-01704-0.

## Background

Obstructive sleep apnea (OSA) is a disorder characterised by repetitive upper airway obstruction during sleep, that might lead to intermittent hypoxia, sleep fragmentation and daytime symptoms such as excessive sleepiness, the so-called obstructive sleep apnea syndrome (OSAS) [1]. Important factors in the progression of the disease include: baseline obesity, older age and the presence of snoring [1].

Compared with persons without sleep apnea, patients with obstructive sleep apnea have heightened peripheral chemoreflex sensitivity, resulting in an increased ventilatory response during hypoxemic episodes [2]. Chemoreflex-mediated vascular sympathetic activation and vasoconstriction intensify as apnea progresses, in association with increases in blood pressure [3]. Increased sympathetic activity, repetitive rises in blood pressure and apnea-induced stress in OSA may also contribute as a trigger to release several prohormones, such as the pro-atrial natriuretic peptide (proANP), the pro-adrenomedullin (proADM) and the pro-arginine vasopressin (proAVP or copeptin) [4, 5].

ProADM is a stable mid-regional fragment that is produced after degradation of ADM [6]. ProADM has been found to be increased in hypoxemia and to have an anti-inflammatory effect on bronchial epithelial cells and airway smooth muscle [7]. ADM counteracts with the renin–angiotensin–aldosterone system [8] and stimulates angiogenesis [9, 10]. Moreover, oxidative stress [11, 12], inflammation [13] and apoptosis [14–16] can induce ADM synthesis. All of these stimuli might be active in the case of OSA.

Arginine vasopressin (AVP) is one of the key hormones of water homeostasis and is produced by hypothalamic neurons [4]. AVP has vasoconstrictor and antidiuretic properties and can restore vascular tone in vasodilatory hypotension [17]. Its release is induced by different stimuli including hypotension, hypoxia, hyperosmolarity, acidosis, infections and various cytokines [18]. AVP is derived from a larger precursor (preproAVP) along with two other peptides: neurophysin II and copeptin [19]. Copeptin is a more stable peptide and its concentrations mirror that of AVP.

Atrial natriuretic peptide (ANP) is a family member of the natriuretic peptides [4]. Its biological role encompasses natriuresis, vasodilation and inhibition of the renin–angiotensin–aldosterone axis and the sympathetic nervous system [20–22]. ProANP, the precursor ANP hormone, is secreted in an equimolar ratio to ANP. As a result of its longer half-life, plasma levels of proANP are at least ten times higher than those of ANP [23] and due to its more stable nature, proANP is considered to be more applicable in clinical practice [24]. Moreover, ANP plays an important role in the regulation of blood pressure, sodium and volume homeostasis and is secreted into the circulation in response to a variety of stimuli, including acute hypoxia [25] and atrial stretch, which occurs in response to pressure and volume loading [26].

OSAS-induced hypoxia, hemodynamic effects, sympathetic activation, vascular endothelial dysfunction, metabolic dysregulation, oxidative stress as well as inflammation might lead to elevated levels of circulating copeptin, proANP and proADM levels. The objective of this study was to evaluate whether copeptin, proANP and proADM levels are higher in patients with OSA and whether they might change under continuous positive airway pressure (CPAP) therapy, serving as potential proxies for the diagnosis and therapy-response in OSA.

## Methods

### Study design

**Fig. 1:**
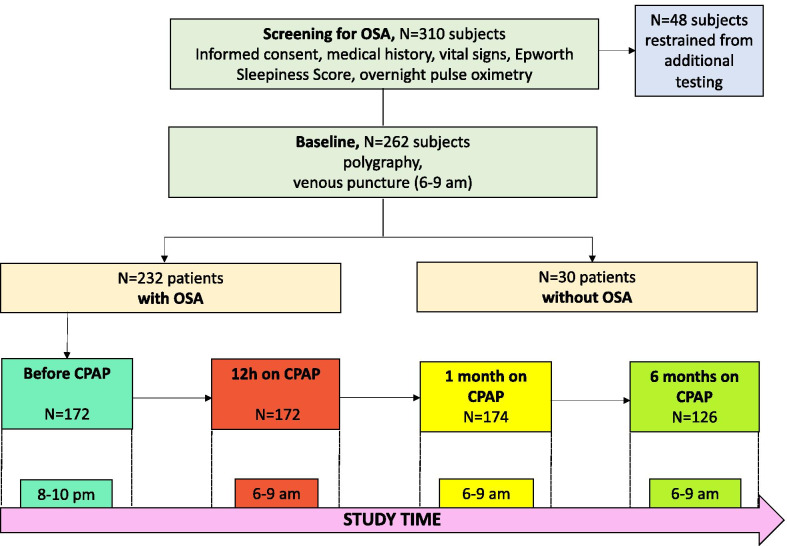
Study design

This is a prospective, observational longitudinal multicenter study. The study was approved by the local Institutional Review Board (EKBB 17010) and was conducted in accordance with the principles of the Helsinki declaration. Ambulatory patients presenting with suspicion of sleep apnea to a respiratory physician, either in private practice or at a tertiary care hospital, during a period of 31 months were considered for inclusion. After providing written informed consent, patients were assessed in terms of relevant medical history and current medical status. Demographics, vital signs, height, weight and the Epworth Sleepiness Scale (ESS) were recorded and screening for OSA was performed using overnight pulse oximetry (Fig. 1). Thereafter, patients underwent polygraphy, followed by a venous puncture in the morning (6–9 a.m.). All patients with OSA spent another night at the center to initiate CPAP therapy. Vital signs were measured and a venous puncture was conducted in the night before CPAP (8–10 p.m.) and in the following morning (6–9 am). In the acquired blood, copeptin, pro-ANP and pro-ADM levels were measured. At one 1 and 6 months of treatment, polygraphy was performed at the center, followed by a venous puncture in the following morning (6–9 a.m.) (Fig. 1, Additional file 1: Table S1).

### Study population

A total of 310 patients with suspicion of OSA were screened for the study. The inclusion criteria were: age ≥ 18 and suspicion of OSA. We excluded patients with mental disorders, which could hinder their judgment concerning study participation, with severe co-morbidities that could result in a lower than 6 months life expectancy, with planned emigration or relocation within the country during the study period, as well as women, who were pregnant or were breast-feeding.

### Examinations

Polygraphy was conducted according to the American Academy of Sleep Medicine recommendations [27] using a commercially available device (WatchPAT™200 Unified, Itamar medical). The following parameters were measured via three points of contact: peripheral arterial tonometry signal, heart rate, oximetry, actigraphy, body position, snoring, chest motion, sleep efficiency and sleep stage distribution. Apnea–hypopnea index (AHI), respiratory disturbance index and oxygen desaturation index (ODI) based on true sleep time and sleep staging were assessed. Apnea was defined as complete cessation of airflow for ≥ 10 s. Mild sleep apnea was defined as 5 ≤ AHI < 15, moderate sleep apnea as 15 ≤ AHI < 30 and severe sleep apnea as AHI ≥ 30. OSAS was defined as an AHI > 5 with presence of daytime symptoms, such as sleepiness or cognitive deficits attributed to sleep apnea. Patients with an AHI ≥ 5 and an ESS < 10 were diagnosed with OSAS without symptoms of sleepiness, while patients with an AHI ≥ 5 and an ESS ≥ 10 were diagnosed with OSAS with symptoms of sleepiness.

### Laboratory analysis

Copeptin was measured using 50 μl of serum by a sandwich immunoassay (CT-proAVP LIA; BRAHMS AG; Hennigsdorf/Berlin, Germany) [28]. The lower detection limit of the assay was 1.7 pmol/l, and the functional assay sensitivity (defined as the lowest value with an intraassay coefficient of variation < 20%) was 2.25 pmol/l [28]. ProANP measurements were performed in 50 μl of serum using a test based on time-resolved amplified cryptate emission (TRACE) technology (MR-proANP KRYPTOR; BRAHMS AG; Hennigsdorf, Germany) [29]. The lower detection limit of the assay was 6 pmol/l and the functional assay sensitivity was 23 pmol/l. ProADM was detected in 50 μl of serum using a sandwich immunoassay (MR-proADM, BRAHMS AG, Hennigsdorf, Berlin, Germany) [30]. The assay had an analytical detection limit of 0.08 nmol/l and a functional assay sensitivity of 0.12 nmol/l.

### Statistical analysis

The results are presented as the mean (standard deviation) for all variables that were normally distributed, and as the median (25th to 75th percentile) for variables that were not normally distributed. Differences between groups were evaluated using the Mann–Whitney U, Kruskal Wallis and Fisher’s Exact tests. Paired comparison and outcome measurements between different visits during the study were examined using the Wilcoxon sign rank test. Correlations between biomarker levels and patient characteristics were performed using the Spearman’s rank correlation.

Single variate associations between prohormone levels at baseline and patient diagnostic characteristics were examined using mixed linear regression models. The multivariate associations between biomarker levels at baseline and patient diagnostic characteristics (those with p < 0.05 in single variate associations) were examined by inclusion of all relevant factors in the model, checking for multi-collinearity. No sign of multi-collinearity was observed for any biomarker (tolerance > 0.10, Variance inflation < 10). Moreover, the associations of prohormone levels with AHI, ESS and ODI severity scores were conducted for all visits utilizing mixed linear models. In all models, the visit factor was included in the model as a fix effect and the subject factor as a random effect. For non-parametric variables we reported the adjusted geometric mean and the effect as the 10^Beta^. All tests were two-tailed; p < 0.05 was defined as significant.

### Sample size calculation

Sample size was calculated using the decrease in median circulating copeptin after 1 month of CPAP treatment as the primary end-point. A sample size of 270 participants was expected to achieve a statistical power of 95% to detect an absolute decrease of copeptin of 25% (from 10.55 to 7.91 considering a standard deviation of 8.5) after 1 month of treatment. Considering a 10% lost to follow-up, we included 310 patients in the study.

## Results

### Patient characteristics

310 patients were included in the study. A total of 48 patients restrained from additional testing after pulsoxymetry, leading to the participation of 262 patients. In 30 of them, OSA could be excluded. Out of the 232 patients with OSA, 116 (50%) of them reported daytime sleepiness (ESS ≥ 10). Moreover, the patients were very well distributed among the OSA severity levels, based on their AHI score, as 86 (37.1%) patients had mild OSA, 78 (33.6%) had moderate OSA, and 68 (29.3%) severe OSA. The average daily use of CPAP during the first therapeutic night was 7.5 (6.3–8.8) hours (AHI: 13.2 ± 11.1), during the first month on CPAP: 5.0 (3.5–6.5) hours (AHI: 4.5 ± 9.1) and during the first six months on CPAP: 5.0 (3.3–6.0) hours (AHI: 3.6 ± 5.9). The majority of the participants were good responders, as only 6 patients deteriorated despite being on CPAP therapy, presenting with an increase in their AHI score in 1 month (n = 2 patients) and 6 months of therapy (n = 4 patients), as compared to the AHI score of the previous visit. The descriptive characteristics of the patients are depicted in Table 1.

**Table 1:** Baseline descriptive characteristics of the patients included in the study

Characteristics	Patients with OSA				Patients without OSA	P-value^$^
	All (N = 232)	Mild OSA (N = 86)	Moderate OSA (N = 78)	Severe OSA (N = 68)	N = 30	
		5 ≤ AHI < 15	15 ≤ AHI < 30	AHI ≥ 30		
Age (mean ± SD)	54.9 ± 13.1	51.5 ± 14.4	54.8 ± 10.3	58.3 ± 13.6	47.9 ± 13.1	0.004^#^
Gender [N, (% male)]	182 (78.4)	61 (70.9)	63 (80.8)	58 (85.3)	15 (50)	0.001^§^
Pack years (mean ± SD)	17.3 ± 24.4	12.8 ± 17.9	16.1 ± 22.9	22.6 ± 31.0	14.6 ± 15.1	0.675^#^
BMI (mean ± SD)	31.3 ± 5.7	29.9 ± 5.6	31.2 ± 5.1	32.5 ± 6.6	26.5 ± 4.6	< 0.001^#^
ODI (mean ± SD)	18.1/h ± 15.3	8.1/ h ± 5.9	18.3/h ± 12.0	41.1 ± 18.1	2.3/h ± 2.2	< 0.001^#^
AHI (mean ± SD)	25.8/h ± 19.0	10.28/h ± 2.90	20.81/h ± 4.19	49.56/h ± 17.31	2.6/h ± 1.4	< 0.001^#^
Treatment*						
Clopidogrel, Prasugrel	46/224 (20.5%)	12/75 (16.0%)	15/74 (20.3%)	16/67 (23.9%)	2/30 (6.7%)	0.082^§^
Diuretic	40/223 (17.9%)	10/75 (13.3%)	12/73 (16.4%)	17/67 (25.4%)	3/30 (10.0%)	0.436^§^
Statin	62/225 (27.5%)	13/75 (17.3%)	22/74 (29.7%)	26/68 (38.2%)	3/30 (10.0%)	0.044^§^
ACE I	85/222 (38.3%)	24/74 (32.4%)	26/73 (35.6%)	33/67 (49.3%)	5/30 (16.7%)	0.025^§^
Calcium Antagonists	25/220 (11.4%)	5/74 (6.8%)	11/72 (15.3%)	7/66 (10.6%)	2/30 (6.7%)	0.752^§^
Beta Blocker	52/222 (23.4%)	13/75 (17.3%)	18/73 (24.7%)	19/66 (28.8%)	3/29 (10.3%)	0.151^§^
Oral Antidiabetic	33/224 (14.7%)	5/75 (6.7%)	10/74 (13.5%)	16/67 (23.9%)	1/30 (3.3%)	0.147^§^
Insulin	6/224 (2.7%)	1/75 (1.3%)	3/74 (4.1%)	2/67 (3.0%)	1/30 (3.3%)	0.590^§^
Anti-depressive	37/224 (16.5%)	16/75 (21.3%)	11/74 (14.9%)	8/67 (11.9%)	6/30 (20.0%)	0.609^§^
Sedatives	23/222 (10.4%)	9/74 (12.2%)	7/73 (9.6%)	5/67 (7.5%)	8/30 (26.7%)	0.018^§^
Comorbidities*						
Cerebrovascular disease	14/229 (6.1%)	7/76 (9.2%)	3/77 (3.9%)	4/68 (5.9%)	1/30 (3.3%)	0.706^§^
Heart failure	12/239 (5.2%)	2/76 (2.6%)	1/78 (1.3%)	8/67 (11.9%)	1/30 (3.3%)	0.722^§^
Myocardial infarction	10/230 (4.4%)	1/76 (1.3%)	4/78 (5.1%)	4/68 (5.9%)	1/30 (3.3%)	1.000^§^
Gastric ulcer	10/230 (4.4%)	1/76 (1.3%)	4/78 (5.1%)	5/68 (7.4%)	1/30 (3.3%)	1.000^§^
Arterial hypertension	108/230 (47.0%)	28/76 (36.8%)	39/78 (50.0%)	38/68 (55.9%)	7/30 (23.3%)	0.018^§^
Coronary artery disease	20/230 (8.7%)	4/76 (5.3%)	6/78 (7.7%)	9/68 (13.2%)	1/30 (3.3%)	0.484^§^
Alcohol dependence	10/230 (4.4%)	2/76 (2.6%)	2/78 (2.6%)	5/68 (7.4%)	1/30 (3.3%)	1.000^§^
COPD	10/230 (4.4%)	1/76 (1.3%)	2/78 (2.6%)	6/68 (8.8%)	0/30 (0%)	0.611^§^
Allergy	37/229 (16.2%)	18/75 (24.0%)	9/78 (11.5%)	8/68 (11.8%)	6/30 (20.0%)	0.603^§^
Diabetes mellitus	40/230 (17.4%)	8/76 (10.5%)	13/78 (16.7%)	17/68 (25.0%)	4/30 (13.3%)	0.796^§^
Sleep characteristics *						
Snoring	211/225 (93.7%)	68/75 (90.7%)	74/77 (96.1%)	61/65 (93.8%)	23/30 (76.7%)	0.006^§^
Breathing pauses	150/222 (67.6%)	48/74 (64.9%)	52/75 (69.3%)	48/65 (73.8%)	14/28 (50.0%)	0.090^§^
Insomnia	39/222 (17.6%)	16/77 (20.8%)	10/73 (13.7%)	12/64 (18.8%)	10/30 (33.3%)	0.050^§^
Restless legs	23/219 (10.0%)	7/74 (9.5%)	8/73 (11.0%)	7/64 (10.9%)	5/28 (17.9%)	0.336^§^
Nycturia	101/221 (45.7%)	36/75 (48.0%)	31/75 (41.3%)	30/63 (47.6%)	9/30 (30.0%)	0.119^§^
Headache	40/217 (18.4%)	14/73 (19.2%)	15/74 (20.3%)	11/63 (17.5%)	8/27 (29.6%)	0.198^§^
ESS [median, (IQR)]	10.0 (6.0–13.0)	9.0 (6.0–13.0)	9.0 (6.0–13.0)	11.5 (5.75–13.25)	12.0 (5.0–14.0)	0.215^#^
Sleep duration (hours) (mean ± SD)	7.4 ± 1.5	7.2 ± 1.5	7.3 ± 1.6	7.5 ± 1.5	7.3 ± 1.7	0.770^#^
Sleep latency (min)	15.6 ± 19.5	16.8 ± 21.2	15.9 ± 21.3	13.7 ± 15.7	35.4 ± 60.9	0.061^#^

*OSA* Obstructive Sleep Apnea, *SD* Standard Deviation, *N* Number, *BMI* Body Mass Index, *ODI* Oxygen Desaturation Index, *AHI* Apnea Hypopnea Index, *ACE I* Angiotensin Converting Enzyme Inhibitors, *COPD* Chronic Obstructive Pulmonary Disease, *ESS* Epworth Sleepiness Scale, *IQR* Interquartile Range, *min* minutes

* Missing data

^$^Comparisons were made between all patients with OSA (N = 232) and patients without OSA (N = 30)

^#^Calculated using the Mann–Whitney U test

^§^Calculated using the Fisher’s Exact test

### Prohormone levels as biomarkers for the diagnosis of OSA

**Fig. 2:**
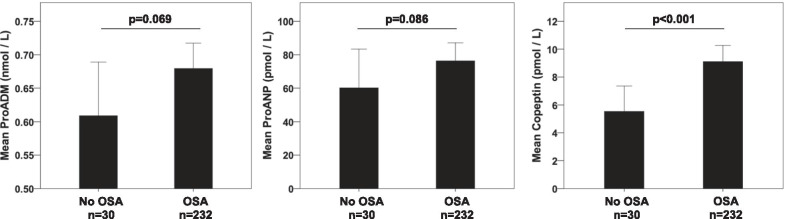
Differences at biomarker levels among patients with and without OSA at baseline. Error bars represent 95% Confidence Intervals. P-values were calculated using the Mann-Whitney U test

When we compared the levels of the three prohormones among patients with and without OSA at baseline, our results indicated that copeptin levels were significantly higher in OSA patients, but there was no significant difference in the levels of proADM (p = 0.069) or proANP (p = 0.086, Fig. 2). Even after 6 months of CPAP therapy, copeptin levels were significantly higher in patients with OSA when compared to the levels of patients without OSA, but there was no significant difference in the levels of proADM and proANP (Table 2).

**Table 2:** Levels of copeptin, proANP and proADM in patients with OSA on CPAP treatment for 6 months and in patients without OSA

	6 months CPAP (N = 100)	No OSA (N = 26)	p-value (Kruskal–Wallis test)
proADM (nmol/l)	0.60 (0.50–0.70)	0.56 (0.50–0.70)	0.420
proANP (pmol/l)	58.38 (40.73–98.74)	47.36 (31.92–69.88)	0.118
Copeptin (pmol/l)	7.95 (4.56–11.48)	4.80 (2.93–7.13)	0.001

**Fig. 3:**
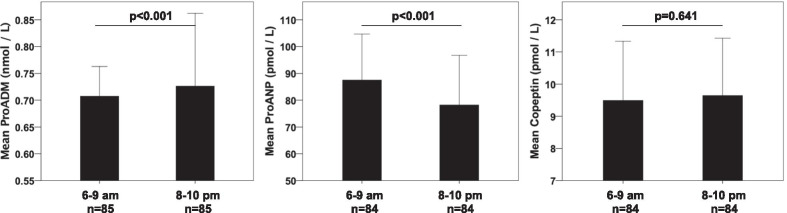
Circadian variation of biomarker levels in patients with OSA between morning (6–9 a.m.) and evening (8–10 p.m.) hours. Error bars represent 95% Confidence Intervals. P-values were calculated using the Wilcoxon signed-rank test

When we compared the levels of the three prohormones in the morning (6–9 a.m., at baseline) and evening (8–10 p.m., before CPAP initiation) in patients with OSA, there was a circadian change in proADM levels, which increased in the evening and in proANP levels, which decreased in the evening (Fig. 3).

Additionally, within the OSA patient group, our results indicated that there was no difference in the levels of proADM [0.62 (0.54–0.73) vs 0.62 (0.55–0.76), p = 0.503], proANP [55.76 (40.00–83.78) vs 54.81 (43.21–90.91), p = 0.998] and copeptin [8.12 (5.30–11.18) vs 7.17 (4.73–10.46), p = 0.207] among patients with and without OSAS, respectively. Additionally, there was no difference in the level of the three prohormones among patients with mild, moderate and severe OSA, as assessed by the AHI, at baseline (Table 3). These differences remained non- significant, even after adjusting for age and gender.

**Table 3:** Levels of proADM, proANP and copeptin in patients with mild, moderate and severe OSA, as assessed by the AHI, at baseline

Baseline Levels	OSA severity			p-value (Kruskal -Wallis Test)
	Mild OSA (N = 86) 5 ≤ AHI < 15	Moderate OSA (N = 78) 15 ≤ AHI < 30	Severe OSA (N = 68) AHI ≥ 30	
proADM (nmol/l)	0.60 (0.50–0.74)	0.62 (0.55–0.75)	0.63 (0.59–0.79)	p = 0.222
proANP (pmol/l)	55.34 (42.61–79.64)	54.81 (40.3–85.05)	61.04 (44.77–98.37)	p = 0.672
Copeptin (pmol/l)	7.15 (4.57–10.37)	7.37 (5.3–9.64)	7.92 (5.08–12.81)	p = 0.375

The baseline levels of all three biomarkers were significantly associated with pack-years, number of comorbidities and medications (Additional file 1: Table S2). Moreover, proANP and proADM were associated with age, while on the other hand copeptin and proADM were associated with gender, BMI and neck circumference. Similarly, proADM and proANP levels were significantly associated with nocturia (Additional file 1: Table S2). Our results further indicated that proANP and proADM levels at baseline correlated with mean saturation (rho = − 0.221, p = 0.008 and rho = − 0.312, p < 0.001 accordingly, Table 4). ProADM levels at baseline were correlated with duration of hypoxemia (rho = 0.297, p < 0.001), periodic limb movements of sleep (rho = 0.259, p = 0.014), sleep efficiency (rho = − 0.250, p = 0.015) and BMI (rho = 0.336, p < 0.001), while copeptin was significantly correlated with sleep latency (rho = − 0.216, p = 0.035, Table 4)

**Table 4:** Correlations between biomarker levels at baseline and various sleep parameters in patients with OSA

Correlation of	With	N	rho	p-value
Copeptin	ODI	142	0.105	0.214
proANP		142	− 0.054	0.526
proADM		142	0.101	0.233
Copeptin	Oxygen saturation at rest	141	− 0.133	0.116
proANP		141	− 0.095	0.261
proADM		141	− 0.053	0.535
Copeptin	Mean saturation	144	− 0.146	0.080
proANP		144	− 0.221	0.008
proADM		144	− 0.312	< 0.001
Copeptin	Hypoxemia duration	139	0.105	0.218
proANP		139	0.115	0.177
proADM		139	0.297	< 0.001
Copeptin	AHI	145	0.153	0.067
proANP		145	0.077	0.359
proADM		145	0.154	0.064
Copeptin	PLMI	90	0.045	0.677
proANP		90	0.120	0.261
proADM		90	0.259	0.014
Copeptin	Sleep latency	95	− 0.216	0.035
proANP		95	− 0.052	0.618
proADM		95	0.006	0.951
Copeptin	Sleep efficiency	94	0.198	0.055
proANP		94	− 0.155	0.135
proADM		94	− 0.250	0.015
Copeptin	VAS-general well being	70	0.133	0.271
proANP		70	0.033	0.789
proADM		70	0.054	0.659
Copeptin	ESS	144	0.010	0.906
proANP		144	0.057	0.498
proADM		144	− 0.033	0.692
Copeptin	BMI	143	0.156	0.063
proANP		143	− 0.103	0.219
proADM		143	0.336	< 0.001

*ODI* Oxygen Desaturation Index, *AHI* Apnea Hypopnea Index, *PLMI* Periodic Limb Movements of Sleep, *VAS* Visual Analogue Scale, *ESS* Epworth Sleepiness Scale, *BMI* Body Mass Index, *rho* Spearman Correlation coefficient

### Comparison of prohormone levels in patients with OSA before and on CPAP therapy

In patients with OSA, the levels of proADM changed after 1 month on CPAP therapy when compared to baseline (p < 0.001) and decreased after 6 months on CPAP therapy (p = 0.020, Table 5). Additionally, proANP levels decreased after 12 h on CPAP therapy, when compared to baseline (p < 0.001, Table 5). Those changes were significant, irrespective of disease severity (as assessed by the AHI and ODI scores) and the existence of symptoms (as assessed by the ESS score, Additional file 1: Table S3). The levels of copeptin did not significantly change on CPAP treatment compared to baseline (Table 5). Adjustments for all comorbidities, time on CPAP and efficacy of treatment (as assessed by the AHI and the ESS) did not change these results. Exclusion of the 12 OSA and 1 control subjects with heart failure did not change reported results on proADM and proANP.

**Table 5:** Differences in copeptin, proANP and proADM levels in patients with OSA between different visits

Paired comparison	Number of pairs	Mean difference ± SD of the difference	p-value (Wilcoxon signed rank test)
Copeptin baseline vs 12 h CPAP	82	− 0.59 ± 2.98	0.139
Copeptin baseline vs 1 month CPAP	101	− 0.02 ± 4.13	0.551
Copeptin baseline vs 6 months CPAP	70	0.44 ± 4.60	0.428
proADM baseline vs 12 h CPAP	82	0.01 ± 0.08	0.114
proADM baseline vs 1 month CPAP	102	0.03 ± 0.62	< 0.001
proADM baseline vs 6 months CPAP	72	− 0.04 ± 0.12	0.020
proANP baseline vs 12 h CPAP	82	− 7.19 ± 19.18	< 0.001
proANP baseline vs 1 month CPAP	103	1.68 ± 27.10	0.504
proANP baseline vs 6 months CPAP	72	2.82 ± 30.95	0.277

## Discussion

In this study, we hypothesised that proANP, proADM and copeptin levels are significantly higher in patients with OSA, when compared to patients without OSA. Our results indicated that only copeptin levels differed significantly among patients with and without OSA at baseline, but also 6 months after CPAP therapy. We found a significant circadian variation in the levels of proADM, which increased in the evening and proANP, which decreased in the evening. In patients with OSA, the levels of proADM significantly changed after 1 and 6 months on CPAP therapy, when compared to baseline. Additionally, proANP levels significantly decreased after 12 h on CPAP therapy, as compared to baseline levels. Those changes were significant irrespective of disease severity and the existence of symptoms.

The current findings come in partial agreement with the results of Cinarka et al. [31] who demonstrated that copeptin levels were higher in 116 patients with OSA, when compared to 27 controls. However, they also proposed that copeptin levels in OSA patients with AHI ≥ 30 were significantly higher when compared to OSA patients with AHI < 30 and that copeptin levels can be used as a predictor for severe OSA, being weakly correlated with AHI, ODI, arousal index and CRP. Our results, conversely, indicated that copeptin levels are significantly correlated with neither AHI nor ODI and that copeptin levels are not significantly different among patients with mild, moderate and severe OSA. This comes in agreement with the study of Selçuk et al. [32], in which there was no statistically significant difference in the levels of copeptin among different OSA severity groups based on AHI scores in 59 OSA patients. The discrepancies to the study of Cinarka et al. [31] might be attributed to specific patient characteristics in both studies as well as the lack of information about whether patients included in the Cinarka study were on CPAP therapy at the time when copeptin levels were measured [31]. Regarding the impact of CPAP therapy in copeptin levels, our results indicate that after 6 months of CPAP therapy, copeptin levels did not reduce significantly when compared to baseline levels and remained significantly different from the control group. This is in agreement with the study of Osma et al. [33], in which serum copeptin levels did not decrease significantly in 16 patients with severe OSA (AHI > 30) after 1 year of positive airway pressure treatment (p = 0.156). This might indicate that copeptin levels cannot be used as a proxy for the response to CPAP therapy. Yet, in the study of Osma et al. there was no significant difference of copeptin levels between OSA patients (N = 16) and the control group (N = 20, p = 0.077) and this discrepancy to our study might be attributed to the very small sample number of the study of Osma et al. [33].

We found a circadian rhythm in the levels of proADM, which increased in the evening and in the levels of proANP which decreased in the evening. Indeed, in previous studies proADM and proANP demonstrated circadian variations [34–36]. However, copeptin did not present with a circadian variation, which is in line with the study of Darzy et al. [37].

Conflicting results exist about the effects of CPAP therapy on plasma proADM levels in OSA patients. In a study by Schulz et al. including 41 OSA patients and 28 controls without sleep-disordered breathing, OSA patients had markedly elevated ADM concentrations at baseline when compared to the controls [38]. After two nights of CPAP therapy, in 28 OSA patients ADM levels significantly decreased when compared to pre-CPAP values, and after 8 months of CPAP treatment, in 11 OSA patients, ADM levels further declined to levels similar with the controls [38]. On the other hand, in a small study including 15 OSA patients and 10 controls, Wolk et al. could not find any difference between ADM levels at baseline and ADM levels after 4 h of CPAP therapy [39]. In our study, we included a larger number of patients and controls and demonstrated that there was no significant difference in proADM levels among patients with and without OSA and that in patients with OSA, proADM levels significantly changed after 1 month on CPAP therapy and significantly decreased after 6 months on CPAP therapy, when compared to baseline. Adjustments for all comorbidities, hours on CPAP and efficacy of treatment (as assessed by the AHI and the ESS on CPAP) did not change these results. ADM is secreted from various organs and is mainly produced by vascular endothelial cells, playing an important role as a vasodilator, positive inotropic, diuretic, natriuretic and bronchodilator [40, 41]. Several stimuli, such as hypoxia, shear stress and inflammatory cytokines can induce proADM production and all these stimuli are increased in patients with OSA [42]. Moreover, it has been identified as a prognostic marker able to stratify mortality risk in sepsis patients with different degrees of organ failure [43], to predict all-cause mortality in pulmonary embolism [44] and increased complications and higher mortality rates in patients suffering from community acquired pneumonia [45]. OSA-induced hypoxic stress and oxidative stress increase circulating inflammatory mediators, including adhesion molecules, inflammatory cytokines and C-reactive protein, leading to hypertension and cardiovascular events [46, 47]. This stress and the related inflammatory molecules are implicated in the production of adrenomedullin which is a potent vasodilator [48]. Adrenomedullin has also been associated with the magnitude of oxyhemoglobin desaturation in OSA patients and with production of reactive oxygen species by leukocytes and treatment with nasal CPAP reduced these parameters in patients with OSA [49]. Therefore, it seems that the upregulation of proADM in OSA constitutes an adaptive counteractive mechanism to protect against cardiovascular diseases in patients with OSA. The reversal of the pathophysiological mechanisms of OSA by implementing non-invasive ventilation might in turn explain the longitudinal decrease of ProADM observed in patients on CPAP therapy.

Previous studies investigating ANP levels in OSA demonstrated that treatment with CPAP acutely reduces plasma ANP levels [36, 50]. We also demonstrated that in 82 patients with OSA, proANP levels significantly decreased after 12 h on CPAP therapy, as compared to baseline levels. Those changes were significant irrespective of disease severity and the existence of symptoms. That comes, nonetheless, in contrast with the results of Mackay et al. who demonstrated that in 9 patients with OSAS, treatment with CPAP for 2 days did not decrease plasma ANP levels [51]. Nevertheless, the study of Mackay et al. included a very small number of patients, when compared to our study. Other studies, could not find any association between OSA and plasma ANP levels [52–54] or even found inverse relations between plasma ANP and AHI [51]. Similarly, in our study, proANP levels were not significantly different between patients with and without OSA, and furthermore, proANP levels were not significantly associated with the severity of OSA, as assessed by the AHI.

A potential limitation of our study is the relatively short follow-up period. A longer term of treatment would have provided a better insight to the effect of CPAP on the prohormone levels. However, compared to previous studies [38, 39, 51–54], this follow up period was the longest. Moreover, the sample size was estimated based on changes in copeptin levels and thus a beta error for proADM and proANP levels cannot be excluded. Nevertheless, no other study so far has investigated proADM and proANP level changes in such a large group of OSA patients. Another limitation of our study is that we utilized only standardised diagnostic and therapeutic methods for OSA and that prohormone levels were measured only in five different time points and not hourly. Yet, to our knowledge, this is the first study that has investigated proADM, proANP and copeptin levels in various time points in a large group of fully characterised OSA patients.

## Conclusion

The results of our study indicate that copeptin is significantly associated with the presence of OSA. None of the three biomarkers reflects disease severity. ProANP serum levels might serve as a potential proxy for the acute response to non-invasive ventilation (12 h), while proADM reflects the long-term response to CPAP therapy (1 and 6 months). This is the first trial that has investigated proADM, proANP and copeptin levels in various time points in a large group of fully characterized OSA patients and the results are of great importance for sleep and respiratory physicians.

## Supplementary Information

**Additional file 1.** **Table S1**. Diagram of the examinations/interventions planned during the study. **Table S2**. Mixed linear models for the association of biomarker levels at baseline and diagnostic characteristics for obstructive sleep apnea. **Table S3**. Mixed linear model for the changes of biomarker levels in different visits, taking into consideration OSA severity, as assessed by the AHI, ESS and ODI scores.

## Data Availability

The datasets used and analysed during the current study are available from the corresponding author on reasonable request.

## Abbreviations

AHI: Apnea–hypopnea index
ANP: Atrial natriuretic peptide
AVP: Arginine vasopressin
CPAP: Continuous positive airway pressure
ESS: Epworth sleepiness scale
ODI: Oxygen desaturation index
OSA: Obstructive sleep apnea
OSAS: Obstructive sleep apnea syndrome
proADM: Pro-adrenomedullin
proANP: Pro-atrial natriuretic peptide
proAVP: Pro-arginine vasopressin

## Declarations

### Guarantor statement

D. Stolz takes full responsibility for the content of the manuscript, including the data and the analysis thereof.
