# Supplementary material for: Copeptin, pro-atrial natriuretic peptide and pro-adrenomedullin as markers of hypoxic stress in patients with obstructive sleep apnea—a prospective intervention study
Source: Respir Res. 2021 Apr 20;22:114. doi: 10.1186/s12931-021-01704-0 (PMC8059312; doi:10.1186/s12931-021-01704-0)
Supplement: Supplementary file 1 — Additional file 1. Table S1. Diagram of the examinations/interventions planned during the study. Table S2. Mixed linear models for the association of biomarker levels at baseline and diagnostic characteristics for obstructive sleep apnea. Table S3. Mixed linear model for the changes of biomarker levels in different visits, taking into consideration OSA severity, as assessed by the AHI, ESS and ODI scores. [file 12931_2021_1704_MOESM1_ESM.docx]

**Copeptin, pro-atrial natriuretic peptide and pro-adrenomedullin as markers of hypoxic stress in patients with obstructive sleep apnea - a prospective intervention trial**

Meropi Karakioulaki^1^, Peter Grendelmeier^1^, Werner Strobel^1^, Thomas Schmid^2^, Kathleen Jahn^1^, Leticia Grize^1^, Michael Tamm^1^, Daiana Stolz^1^

^1^Clinic of Respiratory Medicine and Pulmonary Cell Research, University Hospital of Basel, Switzerland

^2^Privaltklinik Obach, Solothurn, Switzerland

**Corresponding Author**

Prof. Daiana Stolz, MD, MPH, FERS, FCCP

Clinic of Respiratory Medicine and Pulmonary Cell Research

University Hospital Basel

Petersgraben 4, CH-4031 Basel, Switzerland

Email: daiana.stolz@usb.ch

Tel: 0041-61-328 7193

**Supplementary Table 1**: Diagram of the examinations/interventions planned during the study.

|  | **Screening for OSA** | **Before CPAP** | **12h on CPAP** | **1 month on CPAP** | **6 months on CPAP** |
| --- | --- | --- | --- | --- | --- |
| Informed Consent | X |  |  |  |  |
| Medical History | X | X |  |  |  |
| Current medical status | X |  |  | X | X |
| Physical examination |  | X |  | X | X |
| Demographics | X |  |  |  |  |
| Vital Signs | X | X |  | X | X |
| Height and Body weight | X |  |  | X | X |
| Venous puncture (8-10 pm) |  | X |  |  |  |
| Venous puncture (6-9 am) | X |  | X | X | X |
| ESS | X |  | X | X | X |
| Overnight pulse oximetry | X | X |  | X | X |
| Polygraphy | X |  |  |  |  |

**ESS=** Epworth Sleepiness Score

**Supplementary Table 2:** Mixed linear models for the association of biomarker levels at baseline and diagnostic characteristics for obstructive sleep apnea.

| **Outcome** | **Factor** | **Value** | **10^Beta^** | **10^SE^** | **Adjusted geometric mean** | **95% CI for the geometric mean** | **p-Value (Adjusted for multiple comparisons)** |
| --- | --- | --- | --- | --- | --- | --- | --- |
| **Log10 Copeptin** | Age | 1-unit increase | 1.005 | 1.004 |  |  | 0.160 |
|  | Gender | Male  Female | 1.686 | 1.110 | 8.188  4.857 | 7.424-9.031  4.052-5.822 | **<0.001** |
|  | Snoring | No  Yes | 0.922 | 1.203 | 6.836  7.414 | 4.809-9.719  6.727-8.171 | 0.662 |
|  | Breathing pauses | No  Yes | 0.961 | 1.109 | 7.030  7.319 | 5.928-8.338  6.530-8.204 | 0.699 |
|  | Insomnia | No  Yes | 1.148 | 1.133 | 7.525  6.555 | 6.793-8.336  5.233-8.211 | 0.272 |
|  | Restless legs | No  Yes | 0.913 | 1.163 | 7.215  7.903 | 6.520-7.985  5.965-10.471 | 0.548 |
|  | Nocturia | No  Yes | 0.904 | 1.103 | 6.884  7.613 | 6.037-7.850  6.609-8.769 | 0.304 |
|  | Headaches | No  Yes | 1.464 | 1.140 | 7.814  5.337 | 7.034-8.680  4.215-6.758 | **0.004** |
|  | Pack years | 1-unit increase | 1.007 | 1.002 |  |  | **<0.001** |
|  | BMI | 1-unit increase | 1.023 | 1.009 |  |  | **0.009** |
|  | Systolic Blood pressure | 1-unit increase | 0.999 | 1.003 |  |  | 0.572 |
|  | Diastolic Blood pressure | 1-unit increase | 0.996 | 1.004 |  |  | 0.291 |
|  | neck circumference | 1-unit increase | 1.025 | 1.009 |  |  | **0.004** |
|  | Number of comorbidities | 1-unit increase | 1.073 | 1.036 |  |  | **0.048** |
|  | Number of medications | 1-unit increase | 1.057 | 1.026 |  |  | **0.034** |
| **Log10 proANP** | Age | 1-unit increase | 1.030 | 1.003 |  |  | **<0.001** |
|  | Gender | Male  Female | 0.871 | 1.112 | 59.279  68.061 | 53.460-65.721  56.766-81.611 | 0.193 |
|  | Snoring | No  Yes | 1.223 | 1.187 | 73.435  60.062 | 53.113-101.510  54.611-66.066 | 0.242 |
|  | Breathing pauses | No  Yes | 0.946 | 1.108 | 59.047  62.431 | 49.943-69.815  55.769-69.885 | 0.587 |
|  | Insomnia | No  Yes | 1.019 | 1.127 | 60.506  59.402 | 54.668-66.962  48.012-73.497 | 0.878 |
|  | Restless legs | No  Yes | 0.789 | 1.151 | 58.157  73.672 | 52.708-64.166  56.790-95.564 | 0.095 |
|  | Nocturia | No  Yes | 0.705 | 1.096 | 53.481  75.893 | 47.370-60.375  66.425-86.720 | **<0.001** |
|  | Headaches | No  Yes | 1.102 | 1.139 | 62.604  56.820 | 56.440-69.438  44.920-71.888 | 0.458 |
|  | Pack years | 1-unit increase | 1.005 | 1.002 |  |  | **0.007** |
|  | BMI | 1-unit increase | 0.996 | 1.008 |  |  | 0.604 |
|  | Systolic Blood pressure | 1-unit increase | 1.000 | 1.002 |  |  | 0.942 |
|  | Diastolic Blood pressure | 1-unit increase | 0.996 | 1.004 |  |  | 0.338 |
|  | neck circumference | 1-unit increase | 1.000 | 1.009 |  |  | 0.977 |
|  | Number of comorbidities | 1-unit increase | 1.162 | 1.034 |  |  | **<0.001** |
|  | Number of medications | 1-unit increase | 1.126 | 1.024 |  |  | **<0.001** |
| **Log10 proADM** | Age | 1-unit increase | 1.011 | 1.001 |  |  | **<0.001** |
|  | Gender | Male  Female | 0.894 | 1.051 | 0.636  0.711 | 0.606-0.667  0.653-0.775 | **0.025** |
|  | Snoring | No  Yes | 0.989 | 1.084 | 0.646  0.653 | 0.554-0.752  0.624-0.683 | 0.894 |
|  | Breathing pauses | No  Yes | 0.982 | 1.050 | 0.646  0.657 | 0.596-0.699  0.623-0.693 | 0.708 |
|  | Insomnia | No  Yes | 1.029 | 1.057 | 0.653  0.635 | 0.623-0.685  0.575-0.701 | 0.606 |
|  | Restless legs | No  Yes | 0.953 | 1.069 | 0.610  0.721 | 0.575-0.646  0.677-0.769 | 0.465 |
|  | Nocturia | No  Yes | 0.845 | 1.045 | 0.661  0.643 | 0.630-0.695  0.575-0.719 | **<0.001** |
|  | Headaches | No  Yes | 1.029 | 1.064 |  |  | 0.650 |
|  | Pack years | 1-unit increase | 1.003 | 1.001 |  |  | **0.005** |
|  | BMI | 1-unit increase | 1.021 | 1.004 |  |  | **<0.001** |
|  | Systolic Blood pressure | 1-unit increase | 1.000 | 1.001 |  |  | 0.845 |
|  | Diastolic Blood pressure | 1-unit increase | 0.999 | 1.002 |  |  | 0.552 |
|  | neck circumference | 1-unit increase | 1.009 | 1.004 |  |  | **0.032** |
|  | Number of comorbidities | 1-unit increase | 1.096 | 1.015 |  |  | **<0.001** |
|  | Number of medications | 1-unit increase | 1.075 | 1.011 |  |  | **<0.001** |

**Supplementary Table 3:** Mixed linear model for the changes of biomarker levels in different visits, taking into consideration OSA severity, as assessed by the AHI, ESS and ODI scores.

| **Outcome** | **Factor** | **Value** | **p-Value (overall effect of factor)** | **10^Beta^** | **10^SE^** | **Adjusted geometric mean of outcome variable for this level of the factor** | **95%CI (for the geometric mean)** |
| --- | --- | --- | --- | --- | --- | --- | --- |
| **Log 10 Copeptin** | AHI | Normal (<5)  Mild (5-14)  Moderate (15-29)  Severe (≥30) | 0.421 | 0.981  1.066  1.055  *Ref.* | 1.098  1.077  1.076 | 7.458  8.106  8.022  7.603 | 6.676-8.332  7.303-8.997  7.039-9.144  6.466-8.940 |
|  |  |  |  |  |  |  |  |
|  |  |  |  |  |  |  |  |
|  |  |  |  |  |  |  |  |
|  | Visit | Baseline  12h on CPAP  1 month on CPAP  6 months on CPAP | 0.130 | 0.889  0.873  0.893  *Ref.* | 1.081  1.069  1.054 | 7.593  7.452  7.630  8.541 | 6.865-8.398  6.685-8.307  6.723-8.659  7.403-9.853 |
|  |  |  |  |  |  |  |  |
|  |  |  |  |  |  |  |  |
|  |  |  |  |  |  |  |  |
| **Log 10 Copeptin** | ESS | Normal (≤8)  Mild/Mod (9-14)  Severe (15-24) | 0.875 | 1.001  0.976  *Ref.* | 1.079  1.074 | 7.620  7.435  7.617 | 6.882-8.437  6.695-8.257  6.570-8.830 |
|  |  |  |  |  |  |  |  |
|  |  |  |  |  |  |  |  |
|  | Visit | Baseline  12h on CPAP  1 month on CPAP  6 months on CPAP | 0.362 | 0.928  0.896  0.938  *Ref.* | 1.054  1.070  1.050 | 7.464  7.207  7.540  8.039 | 6.793-8.201  6.286-8.263  6.783-8.382  7.133-9.060 |
|  |  |  |  |  |  |  |  |
|  |  |  |  |  |  |  |  |
|  |  |  |  |  |  |  |  |
| **Log 10 Copeptin** | ODI Severity | Normal (<5)  Mild (5-14)  Moderate (15-29)  Severe (≥30) | 0.544 | 0.957  1.010  1.051  *Ref.* | 1.098  1.091  1.094 | 7.496  7.913  8.233  7.835 | 6.805-8.258  7.163-8.741  7.151-9.479  6.547-9.378 |
|  | Visit | Baseline  12h on CPAP  1 month on CPAP  6 months on CPAP | 0.365 | 0.915  0.950  0.921  *Ref.* | 1.064  1.057  1.052 | 7.609  7.898  7.659  8.314 | 6.916-8.371  6.977-8.941  6.810-8.614  7.318-9.445 |
|  |  |  |  |  |  |  |  |
|  |  |  |  |  |  |  |  |
|  |  |  |  |  |  |  |  |
| **Log 10 proANP** | AHI Severity | Normal (<5)  Mild (5-14)  Moderate (15-29)  Severe (≥30) | 0.366 | 1.080  1.030  0.983  *Ref.* | 1.067  1.053  1.051 | 63.267  60.301  57.595  58.565 | 56.970-70.260  54.459-66.769  51.307-64.653  51.211-66.974 |
|  |  |  |  |  |  |  |  |
|  |  |  |  |  |  |  |  |
|  |  |  |  |  |  |  |  |
|  | Visit | Baseline  12h on CPAP  1 month on CPAP  6 months on CPAP | **0.003** | 1.040  0.926  1.016  *Ref.* | 1.055  1.045  1.035 | 62.631  55.754  61.203  60.213 | 56.667-69.223  50.255-61.855  54.634-68.559  53.310-68.010 |
|  |  |  |  |  |  |  |  |
|  |  |  |  |  |  |  |  |
|  |  |  |  |  |  |  |  |
| **Log 10 proANP** | ESS Severity | Normal (≤8)  Mild/Mod (9-14)  Severe (15-24) | 0.779 | 0.967  0.959  *Ref.* | 1.065  1.060 | 59.545  59.068  61.592 | 53.966-65.699  53.438-65.291  53.950-70.316 |
|  |  |  |  |  |  |  |  |
|  |  |  |  |  |  |  |  |
|  | Visit | Baseline  12h on CPAP  1 month on CPAP  6 months on CPAP | **0.016** | 0.977  0.867  1.016  *Ref.* | 1.043  1.055  1.039 | 60.938  54.041  63.358  62.355 | 55.560-66.837  47.860-61.021  57.299-70.059  55.866-69.598 |
|  |  |  |  |  |  |  |  |
|  |  |  |  |  |  |  |  |
|  |  |  |  |  |  |  |  |
| **Log 10 proANP** | ODI Severity | Normal (<5)  Mild (5-14)  Moderate (15-29)  Severe (≥30) | 0.247 | 0.970  0.931  1.022  *Ref.* | 1.074  1.068  1.070 | 61.736  59.275  65.070  63.681 | 56.002-68.057  53.683-65.448  57.370-73.801  54.698-74.138 |
|  |  |  |  |  |  |  |  |
|  |  |  |  |  |  |  |  |
|  |  |  |  |  |  |  |  |
|  | Visit | Baseline  12h on CPAP  1 month on CPAP  6 months on CPAP | **0.005** | 0.972  0.905  1.027  *Ref.* | 1.048  1.041  1.037 | 62.223  57.925  65.723  64.010 | 56.497-68.530  51.651-64.961  58.864-73.381  56.999-71.884 |
|  |  |  |  |  |  |  |  |
|  |  |  |  |  |  |  |  |
|  |  |  |  |  |  |  |  |
| **Log 10 proADM** | AHI Severity | Normal (<5)  Mild (5-14)  Moderate (15-29)  Severe (≥30) | 0.969 | 1.008  1.016  1.015  *Ref.* | 1.052  1.041  1.041 | 0.658  0.663  0.663  0.653 | 0.619-0.699  0.626-0.703  0.617-0.712  0.597-0.713 |
|  |  |  |  |  |  |  |  |
|  |  |  |  |  |  |  |  |
|  |  |  |  |  |  |  |  |
|  | Visit | Baseline  12h on CPAP  1 month on CPAP  6 months on CPAP | **0.014** | 1.067  1.117  1.040  *Ref.* | 1.043  1.037  1.029 | 0.667  0.698  0.650  0.625 | 0.631-0.705  0.657-0.741  0.606-0.696  0.578-0.676 |
|  |  |  |  |  |  |  |  |
|  |  |  |  |  |  |  |  |
|  |  |  |  |  |  |  |  |
| **Log 10 proADM** | ESS Severity | Normal (≤8)  Mild/Mod (9-14)  Severe (15-24) | 0.154 | 0.919  0.938  *Ref.* | 1.045  1.042 | 0.636  0.650  0.693 | 0.603-0.671  0.615-0.687  0.638-0.752 |
|  |  |  |  |  |  |  |  |
|  |  |  |  |  |  |  |  |
|  | Visit | Baseline  12h on CPAP  1 month on CPAP  6 months on CPAP | 0.155 | 1.053  1.096  1.040  *Ref.* | 1.032  1.042  1.030 | 0.663  0.690  0.655  0.630 | 0.631-0.696  0.639-0.746  0.619-0.693  0.590-0.672 |
|  |  |  |  |  |  |  |  |
|  |  |  |  |  |  |  |  |
|  |  |  |  |  |  |  |  |
| **Log 10 proADM** | ODI Severity | Normal (<5)  Mild (5-14)  Moderate (15-29)  Severe (≥30) | 0.110 | 0.894  0.928  0.965  *Ref.* | 1.052  1.049  1.051 | 0.643  0.668  0.694  0.720 | 0.611-0.678  0.633-0.705  0.643-0.750  0.652-0.794 |
|  |  |  |  |  |  |  |  |
|  |  |  |  |  |  |  |  |
|  |  |  |  |  |  |  |  |
|  | Visit | Baseline  12h on CPAP  1 month on CPAP  6 months on CPAP | **<0.001** | 1.027  1.146  1.047  *Ref.* | 1.035  1.031  1.028 | 0.664  0.740  0.676  0.646 | 0.630-0.699  0.692-0.792  0.634-0.721  0.603-0.693 |
|  |  |  |  |  |  |  |  |
|  |  |  |  |  |  |  |  |
|  |  |  |  |  |  |  |  |

**OSA=** Obstructive Sleep Apnea, **AHI=** Apnea Hypopnea Index, **ESS=** Epworth Sleepiness Scale, **ODI=** Oxygen Desaturation Index
